# A case of severe psychosis induced by novel recreational drugs

**DOI:** 10.12688/f1000research.3-21.v1

**Published:** 2014-01-22

**Authors:** Filippo Dragogna, Lucio Oldani, Massimiliano Buoli, A. Carlo Altamura

**Affiliations:** 1 Department of Psychiatry, University of Milan, Fondazione IRCCS Ca’ Granda, Ospedale Maggiore Policlinico, Milan, Italy

## Abstract

**Introduction: ** The use of novel recreational drugs is becoming of public interest, especially after recent international alerts about their cardiovascular and neurological toxicity. Additionally, little is known about the psychiatric consequences of the long-term use of these compounds.

**Case presentation: **We describe a case of severe psychotic episode likely induced by chronic use of a combination of new recreational drugs (methylenedioxypyrovalerone, mephedrone, butylone and alpha-pyrrolidinopentiophenone). The patient had no psychiatric history and showed poor response to conventional antipsychotic treatment (haloperidol).

**Conclusions: **This case illustrates the potential negative effects of recreational drugs that cannot be limited to an acute psychotic episode but might determine a condition of prolonged paranoid psychosis. Although the use of these compounds is currently increasing, such molecules might often pass undetected in patients accessing the emergency room, leading to misdiagnosis (e.g. schizophrenic episode) and lack of appropriate treatment.

## Case description

We report the case of a 46-y.o. Italian Caucasian man (1.70 m, 70 kg), working as a full-time legal consultant.

He was brought to the emergency room of our Psychiatric Department in 2013, after having ingested a large quantity of zolpidem, a prescription drug indicated for insomnia, with suicidal intent. His family history was negative for psychiatric disorders, but he presented a positive medical history for liver disease (active chronic hepatitis C and Gilbert's syndrome). He had never suffered from any psychiatric symptoms in his childhood, adolescence or early adulthood. He had no family history of psychiatric disorders.

The patient’s psychopathological onset occurred three months prior to admission when, after continuous use (from one to three times a week) of a non-specified recreational drug since July 2012, provided to him by a friend who used to buy it online. He developed a persecutory delusion, characterized by the conviction of being spied upon by some unknown people who placed video cameras around his house. In addition, he believed that sexual activity he had with his wife was being filmed and then spread on pornographic websites. For this reason, in the following weeks, he made a written complaint against unknown persons and presented it to the local police office. In addition, one night he called the police force to his own home, as he saw suspicious movements outside his window. On that occasion, the police found out that the patient had a gun, which was then confiscated due to security concerns. Thus, the police then became part of patient’s delusional plot: the patient started thinking his daughter was involved in prostitution and he blamed the police for that. These events, along with two formal warnings received from his employer (since he did not show up for work for many days in a row), and a prolonged condition of global insomnia, generated a state of severe discouragement and embarrassment in the patient. On account of this he attempted suicide by ingesting a large quantity of zolpidem tablets (about 40 tablets of 10 mg, as related by the patient himself). Zolpidem had been prescribed four months prior to admission by a general practitioner to treat the patient’s insomnia.

In the emergency room of our hospital, the patient was initially drowsy and slow in his movements, although he was eupneic and had stable vital parameters. He underwent a physical examination, an electrocardiogram, a chest X-ray and a brain computer tomography, which were all normal. Though initially considered appropriate, no gastric lavage with activated charcoal was conducted as the patient had a spontaneous episode of vomiting. The patient was rehydrated with intravenous physiological saline and then admitted to our ward.

Since the first day of admission, during the daily interviews with the medical staff, the patient showed the same delusional ideas described above and showed a very poor insight about his psychopathological condition [Brief Psychiatric Rating Scale (BPRS) score=50]. He admitted that the drug he had been taking during the previous months might have had a role in the development of his thoughts, but this did not change his belief that he was being persecuted by the police and his colleagues and managers at work. During his hospitalization, which lasted 17 days, his prolonged insomnia improved. He developed a mild criticism of his delusion, doubting some of the events he had reported, but contemporarily maintained a suspicious behavior towards the nurses and the doctors and an overall persecutory ideation. At discharge, he reported that he intended to sue the policeman who visited him at home as he had no right to suspend his gun license, showing overall poor insight and a very solid and structured delusion (BPRS score=31).

As soon as the patient was admitted to our ward, we collected blood and urine samples, which were analyzed by the central laboratory of our hospital. Haematological and chemistry tests highlighted a condition of normocytic anemia (Hb 11.2 mg/dl, Ht 33.4%, mean cell volume (MCV) red cells 3.55 × 10 ^6^/mmc) and a mild hepatic distress [alanine transaminase (ALT) 136 U/l]. Routine screening for psychotropic drugs gave negative results. A positive emission tomography (PET) was performed on the 10 ^th^ day of admission, which showed some unspecific findings consisting of an increased glucose metabolism in the basal nuclei but normal levels in the cortical regions.

During the hospitalization period, a sample of the drug the patient had used for some months, in the form of white powder, was found at his home and delivered to the medical staff. This powder sample, along with further blood and urine samples, were sent to the Legal Medicine laboratory in the Forensic Toxicology section of our University, after having obtained a regular written consent from the patient. A specific analysis on the three samples was conducted by means of gas chromatography/mass spectrometry and liquid chromatography/high resolution mass spectrometry. The following molecules were found by the analysis on the powder: methylenedioxypyrovalerone, mephedrone, butylone and alpha-pyrrolidinopentiophenone (a-PVP) (the proportion of the each was not provided by the laboratory) ( Table 1). Traces of methylenedioxypyrovalerone were found in the urine sample. A test for psychotropic drugs gave negative results for the blood sample.

With regards to the psychopharmacological therapy, since the beginning of his hospitalization the patient was treated with haloperidol 5 mg daily, showing an overall good medication adherence although his response was very poor. For this reason, we decided to administer an injection of haloperidol decanoate at a dosage of 150 mg (to be repeated every 4 weeks, as part of an outpatient regimen). One month after his discharge, in a follow-up visit, the patient showed a BPRS score of 29, corresponding to a slight improvement in persecutory delusion but no change in insight.

**Table 1.** Summary of pharmacological components present in the white powder taken by the patient.

Molecule	Mechanism of action	Effects	Potential risk
**Methylenedioxypyrovalerone**	Norepinephrine-dopamine reuptake inhibitor	**Psychic:** euphoria, increased wakefulness (severe insomnia) **Physical:** tachycardia, hypertension	Hyperthermia ^3, 4^
**Mephedrone**	Serotonin-dopamine reuptake inhibitor, pre-synaptic monoamine release	**Psychic:** elevated mood, hallucinations, delusions, sexual stimulation **Physical:** tachycardia, hypertension, breath depression, increased sweating, teeth grinding	Stroke, heart failure, hyperthermia
**Butylone**	Dopamine reuptake inhibitor, serotonin receptor 2A agonist	**Psychic:** mild euphoria **Physical:** nystagmus, increased body temperature, teeth grinding	Rhabdomyolysis, hyperthermia, acute renal failure
**Alpha-Pyrrolidinopentiophenone** **(a-PVP)**	Norepinephrine-dopamine reuptake inhibitor	**Psychic:** euphoria, anxiety **Physical:** tachycardia, hypertension	Hyperthermia

## Discussion

In contrast to other reports that show hallucinatory delirium, agitation, anxiety and sleep disturbances following an acute intake of more popular substances of abuse (e.g. cannabis, cocaine), we describe a clinical picture of a persistent substance-induced psychosis, secondary to a prolonged intake of methylenedioxypyrovalerone, mephedrone, butylone and alpha-pyrrolidinopentiophenone (a-PVP). The correlation of symptoms with the intake of these substances is supposed in the light of a negative psychiatric history and no concomitant medical treatments. Of note, increased glucose metabolism in the basal nuclei at PET was probably due to the effects of haloperidol than to the substances, as documented by other reports ^1^. In addition, the moderate hepatic failure of the patient could be responsible for the increased availability of the drugs and increased toxicity, as hepatic metabolism is involved in the excretion of all these substances.

The present report shows the danger of these novel drugs that are often bought as apparently safe and legal on the internet. However a number of case reports have documented deaths related to the ingestion of such substances, especially for mephedrone ^2^ and methylenedioxypyrovalerone ^3, 4^. A further problem is that these substances are not detected by standard blood and urine tests so that the diagnosis of intoxication is often delayed. This means that patients may not receive the appropriate treatment due to the lack of diagnostic tools and may be misdiagnosed as schizophrenic or manic bipolar patients. This issue is particularly relevant in the emergency department, where a fast and precise diagnosis and management of patients is required. In addition, patients leaving the emergency department without being correctly diagnosed might continue their abuse and delay specialized follow-up visits in an outpatient context, finally developing a condition of chronic treatment-resistant psychosis.

These drugs are very popular among young clubbers and mephedrone was reported as the sixth most commonly used drug after alcohol, tobacco, cannabis, MDMA and cocaine in the dance music scene, night clubs and gay bars ^5– 7^. Educational campaigns about the risks related to these substances are needed in light of the negative effects that can be sustained over time ^8^. In addition, clinical pictures similar to the one described should not be confused with schizophrenic episodes despite the atypical presentation and good pre-morbid social functioning.

## Consent

Consent was obtained from the patient for use of their information for publication in this article.
