# Supplementary material for: A case of severe psychosis induced by novel recreational drugs
Source: F1000Res. 2014 Jan 22;3:21. [Version 1] doi: 10.12688/f1000research.3-21.v1 (PMC4207243; doi:10.12688/f1000research.3-21.v1)
Supplement: Peer review history

# Peer review: Referee response for version 1

**DOI:** 10.5256/f1000research.3458.r6396

**Published:** 2014-10-13

**Authors:** George Woody

**Affiliations:** 1 Treatment Research Institute, University of Pennsylvania, Philadelphia, USA

This is an interesting case report that is clearly written and documents an instance in which a persistent and treatment resistant psychotic disorder developed after taking an unknown amount of hallucinogenic and stimulant drugs. This case differs from the typical course of substance induced psychoses in that it did not resolve with abstinence and pharmacotherapy. I have no suggestions for modifications.

I have read this submission. I believe that I have an appropriate level of expertise to confirm that it is of an acceptable scientific standard.

# Peer review: Referee response for version 1

**DOI:** 10.5256/f1000research.3458.r3493

**Published:** 2014-03-06

**Authors:** John Mendelson

**Affiliations:** 1 Addiction and Pharmacology Research Laboratory, California Pacific Medical Center Research Institute, San Francisco, CA, USA

Dragogna and colleagues describe a case of persistent psychosis associated with the ingestion of mephadrone and related compounds. These cathinone derivatives have similar risks as other phenethylamine stimulants such as methamphetamine and MDMA and are widely available and easily abused. It is therefore not surprising that persistent psychosis, as described in this case report, can be seen with prolonged use.

Although the authors show an association of cathinone use with admission for a persistent psychosis the relationship between cathinone abuse and the development of psychosis is less clear - this patient may have become psychotic well before using any illicit drugs. However, the case report makes the essential point that urine drug screens with currently available immunoassay are likely to miss cathinone-like drugs. Thus, if cathinone use is suspected, a laboratory work up of biospecimes will need to be performed by groups specializing in medical toxicology. Due to the increasing abuse of drugs in this class clinicians can expect to see more toxicity. Overall, this report adds to the literature.

I have read this submission. I believe that I have an appropriate level of expertise to confirm that it is of an acceptable scientific standard.
